# Gaussian Mixture Cardinalized Probability Hypothesis Density(GM-CPHD): A Distributed Filter Based on the Intersection of Parallel Inverse Covariances

**DOI:** 10.3390/s23062921

**Published:** 2023-03-08

**Authors:** Liu Wang, Guifen Chen, Guangjiao Chen

**Affiliations:** School of Electronic and Information Engineering, Changchun University of Science and Technology, Changchun 130022, China

**Keywords:** distributed fusion, GM-CPHD, parallel inverse covariance intersection, Wave Filter

## Abstract

A distributed GM-CPHD filter based on parallel inverse covariance crossover is designed to attenuate the local filtering and uncertain time-varying noise affecting the accuracy of sensor signals. First, the GM-CPHD filter is identified as the module for subsystem filtering and estimation due to its high stability under Gaussian distribution. Second, the signals of each subsystem are fused by invoking the inverse covariance cross-fusion algorithm, and the convex optimization problem with high-dimensional weight coefficients is solved. At the same time, the algorithm reduces the burden of data computation, and data fusion time is saved. Finally, the GM-CPHD filter is added to the conventional ICI structure, and the generalization capability of the parallel inverse covariance intersection Gaussian mixture cardinalized probability hypothesis density (PICI-GM-CPHD) algorithm reduces the nonlinear complexity of the system. An experiment on the stability of Gaussian fusion models is organized and linear and nonlinear signals are compared by simulating the metrics of different algorithms, and the results show that the improved algorithm has a smaller metric OSPA error than other mainstream algorithms. Compared with other algorithms, the improved algorithm improves the signal processing accuracy and reduces the running time. The improved algorithm is practical and advanced in terms of multisensor data processing.

## 1. Introduction

It is difficult for a single sensor to meet practical needs. Scholars have successively put forward the multisensor information fusion filtering theory, which can divide fusion estimation into two types: centralized fusion and distributed fusion. The advantages of distributed fusion are its robustness and flexibility. The data communication link bandwidth of the distributed fusion sensor is low, and its scalability is satisfactory; moreover, the optimal fusion algorithm can be obtained under the condition that the correlation is known [1,2,3,4,5]. However, in practical applications, the correlation between multisensors is unknown. Without considering multisensor correlation, it is easy for sensor fusion results to diverge. In practical applications, the cross-covariance of multisensors is also unknown. Therefore, the problem of the unknown fusion estimation of the uncertainty time-varying noise variance as well as the cross-covariance of multisensor-distributed fusion warrants further research. In recent years, scholars have proposed a covariance cross-fusion (CI fusion) algorithm for the estimation of multisensor fusion with unknown cross-covariance. The advantage of CI fusion is that it can conservatively combine covariance intersections to make estimations without considering the correlation between nodes. Liu, B. [6] (2017) and Noack, B. [7] (2017) proposed a parallel fusion architecture for distributed- and decentralized-state estimation systems. Hu, Z. [8] (2021) suggested that the enhanced sequence covariance intersection (CI) fusion is feasible, but the unpredictable fusion structure will directly affect the fusion results. Liu, B. [9] (2021) improved the unknown cross-covariance data fusion algorithm, but this algorithm reduced the system’s accuracy. HU, Z. [10] (2022) proposed a feedback CI fusion strategy in their research and applied this method to the local filtering results, but this algorithm has a long operation time and is not suitable for multisensor distributed fusion. Zhang, P. [11] (2022) demonstrated the effectiveness of DEI fusion in their study, but this method is not applicable to multisensor distributed fusion. The advantage of SCI is that it does not need to solve the high-dimensional weight coefficient convex optimization problem. Therefore, relevant scholars have carried out research on this matter, for example, Deng, Z. [12] (2012) suggested in his research that the accuracy of SCI fusion is not very sensitive to the order of the sensors and that the robustness of SCI leans toward BCI fusion. Wang, N. [13] (2020) proposed an SCI-KCF filter in their study. Although this method offers the advantages of stability and limited estimation errors, it consumes a significant amount of time due to the need for multiple ablations, which affects the fusion time. The advantage of BCI fusion is that it can address the mathematical problems in distributed fusion through the average consensus algorithm. Relevant research has been carried out to address this issue, for example, Tao, S. [14] (2016), Liu, Y. [15] (2021), and others proposed a batch covariance intersection (BCI) strategy in their studies to verify the effectiveness of the algorithm, but at the same time, their methods may increase the system’s operation time while also improving the system’s robustness.

In parallel, relevant scholars also carried out relevant research on the application of covariance intersection to the above-mentioned filter. At this stage, covariance intersection is mainly applied to the Kalman filter [16,17,18,19,20,21,22,23,24]. Qi, W. [25] (2020) applied BCI fusion and fast SCI fusion to a time-varying Kalman filter in their research and suggested that this method should solve the high-dimensional nonlinear optimization problem; however, the algorithm’s operation implies great computational complexity and quantity. Kim, S.Y. [26] (2022) proposed an SMC-CPHD filter in their study, which can increase the compensation method of inverse covariance intersection and extend the ICI to multisensor and multitarget systems. Kim (2020) [27] applied ICI fusion to a UKF filter. Chen, H. [28] fused CI into DDUKF-CI. Park, W. J. [29] (2021) applied ICI to a GM-CPHD filter with generalization. The advantage of the inverse covariance intersection (ICI) algorithm is that it can find the most probable public information of the fusion estimation, ensure the consistency of the system, and export the fusion results to remove the boundary of the public information that may be shared [23,30]. Therefore, it can be seen that the application of inverse covariance intersection to distributed sensor fusion is the direction of future development.

In this paper, a GM-CPHD filter based on the parallel inverse covariance intersection is proposed to solve the problem of local filters, uncertain time-varying noise variance, and cross-covariance fusion estimation in multisensor distributed fusion. The GM-CPHD filter is used to filter and estimate the subsystems, and the PICI fusion algorithm is used to fuse the local estimates of each subsystem to improve the solution of high-dimensional weight coefficient convex optimization problems, reduce the running time of the algorithm, and verify the effectiveness of the algorithm through simulation.

## 2. GM-CPHD Filter

A multisensor nonlinear discrete system is expressed as follows:

(1) $$x_{k}=F_{k}x_{k}+B_{k}w_{k}$$

(2) $$z_{k}^{\left(j\right)}=C_{k}^{\left(j\right)}x(k)+v_{k}^{\left(j\right)},j=1,2,\cdots ,L$$

where *L* is the number of sensors, $k\in {\mathbb{R}}^{n\times 1}$ represents discrete time, $x_{k}\in {\mathbb{R}}^{n\times 1}$ expresses the state at time *k*, $F_{k}$ represents a time-varying state-transition matrix with known proper dimensions, $B_{k}$ represents the input noise matrix, $C_{k}^{\left(j\right)}$ represents the observation matrix, $z_{k}^{\left(j\right)}$ represents the measurement of the *j*th sensor at time *k*, $w_{k}\in {\mathbb{R}}^{m\times 1}$ represents the process noise, and $v_{k}^{\left(j\right)}\in {\mathbb{R}}^{m\times 1}$ represents the observation noise of the *j*th sensor, both of which are zero-mean, as follows:

(3) $$E\left\{\left[\begin{array}{l} w_{k}\\ v_{k}^{\left(i\right)} \end{array}\right]\left[\begin{array}{cc} w_{k}^{T} & {\left(v_{i}^{\left(j\right)}\right)}^{T} \end{array}\right]\right\}=\left[\begin{array}{cc} Q_{k} & 0\\ 0 & R_{k}^{\left(j\right)}\delta_{ij} \end{array}\right]\delta_{ij}\;i,j=1,2,\cdots ,L$$

where *E* is the mathematical expectation and *T* is transposed. This function satisfies the following: $\delta_{ii}=1,\delta_{ij}=0(i\neq j)$.

Based on the linear unbiased minimum variance and the minimum maximum estimation, the predictive local filtering of time-varying noise variance can be expressed as follows:

(4) $${\hat{x}}_{k}^{\left(j\right)}=\psi C_{k}^{\left(j\right)}x_{k-1}^{\left(j\right)}+Y_{k}^{\left(j\right)}z_{k}^{\left(j\right)},j=1,2,\cdots ,L$$

The $Y_{k}^{\left(j\right)}$ filter gain is as follows:

(5) $$Y_{k}^{\left(j\right)}=P_{k|k-1}^{\left(j\right)}{\left(C_{k}^{\left(j\right)}\right)}^{T}{\left[C_{k}^{\left(j\right)}P_{k|k-1}^{\left(j\right)}{\left(C_{k}^{\left(j\right)}\right)}^{T}+R_{k}^{h}\right]}^{-1}$$

The estimation error covariance matrix is as follows:

(6) $$P_{k+1|k}^{\left(j\right)}=C_{k}^{h}P_{k|k-1}^{\left(j\right)}{\left(C_{k}^{h}\right)}^{T}+B_{k}Q_{k}{\left(B_{k}\right)}^{T}$$

(7) $$P_{k|k}^{\left(j\right)}=[I-K_{k}^{\left(j\right)}C_{k}^{\left(j\right)}]P_{k|k-1}^{\left(j\right)}$$

where *I* is the unit matrix. In order to ensure the robustness of the system, the local estimation variance matrix should satisfy the Lyapunov equation.

(8) $$\begin{array}{c} P_{k+1|k+1}^{\left(j\right)}=\psi_{k+1}^{\left(j\right)}[I-K_{k+1}^{\left(j\right)}C_{k+1}^{\left(j\right)}]B_{k}Q_{k}{\left(B_{k}\right)}^{T}{\left([I-K_{k+1}^{\left(j\right)}C_{k+1}^{\left(j\right)}]\right)}^{T}\\ +K_{k+1}^{\left(j\right)}R_{k+1}^{\left(j\right)}{\left(K_{k+1}^{\left(j\right)}\right)}^{T}+P_{k|k}^{\left(j\right)}{\left(\psi_{k+1}^{\left(j\right)}\right)}^{T} \end{array}$$

Therefore, the selection of the filter and the fusion effect of the local filter can directly affect the multisensor fusion results, especially for the unknown covariance, unknown motion model, and multisensor distributed fusion with uncertain time-varying noise variance.

### 2.1. CPHD Filter

Common filters include PHD, CPHD, as well as JOINTCPHD and ICPHD filters improved on the basis of the CPHD filter [31,32]. The PHD filter is based on the assumption of Poisson distribution, and its algorithm stability is poor. In order to address this, Vo, B. N. [33,34,35,36] (2007) suggested that the performance of the PHD filter can be improved if the second-order information of the number of targets is introduced comprehensively. It is verified that under the assumption of independent and identical distribution, the tracking accuracy and stability of the CPHD filter are higher, and it can better estimate the performance of the target. Moreover, its adjustability has received great attention and promotion.

The real-value function $RFSX=\{x_{1},\cdots ,x_{a}\}$ and multitarget density function can determine the characteristics of RFS and multitarget density function $RFSX=\{x_{1},\cdots ,x_{a}\}$. The set integration can be defined as follows:

(9) $$\int f(X)\delta X≜\sum_{a=1}^{\infty }\frac{1}{a!}\int f(\{x_{1},\cdots ,x_{a}\})dx_{1}\cdots dx_{a}+f(\phi )$$

The probability generating functional (PGFL) G[b] and probability assumption density D(x) are defined as follows:

(10) $$G[b]≜\int f(X)\prod_{x\in X}b(x)\delta X$$

(11) $$D(x)≜\frac{\delta }{\delta x}G[b]|{}_{b=1}$$

The CPHD filter is characterized by an independent identically distributed process. The basic distribution of point process |X|=a can be assumed to be p(n). Then, G[b] and D(x) can be defined as follows:

(12) $$f(X)≜a!\;\cdot \;p(a)\cdot f(x_{1})\cdots f(x_{a})$$

(13) $$G[b]=\sum_{a=0}^{\infty }p(a){\left(\int h(u)\cdot f(u)du\right)}^{a}$$

(14) $$D(x)≜\frac{\delta }{\delta x}G[b]|{}_{b=1}=f(x)\sum_{a=1}^{\infty }a\cdot p(a)$$

### 2.2. GM-CPHD Filtering

We operate under the following assumptions: (1) The moving target represents a linear Gaussian dynamic model; (2) the detection probability and the survival probability of the moving target have no correlation with the moving state of the target; and (3) the strength of the random set of new targets has the form of a Gaussian mixture. Based on the above assumptions, the recursive form of the GM-CPHD filter can be given as follows [35]:

(1) Prediction: The Gaussian mixture of the posterior intensity at *k* − 1 is

(15) $$v_{k-1}(x)=\sum_{i=1}^{J_{k-1}}w_{k-1}^{\left(i\right)}N(x;m_{k-1}^{\left(i\right)},P_{k-1}^{\left(i\right)})$$

(2) Update: It is assumed that the predicted intensity has the form of a Gaussian mixture, which is

(16) $$v_{k|k-1}(x)=\sum_{i=1}^{J_{k|k-1}}w_{k|k-1}^{\left(i\right)}N(x;m_{k|k-1}^{\left(i\right)},P_{k|k-1}^{\left(i\right)})$$

The function is established according to the standard result of the Gaussian function.

(17) $$\int N(x;Fl+d,Q)N(l;m,P)dl=N(x;F_{m}+d,Q+FPF^{Τ})$$

(18) $$N(z;H_{x},R)N(x;m,P)=q(z)N(x;\tilde{m},\tilde{p})$$

Compared with the GM-CPHD filter, the GM-PHD filter provides recursive closed-form solutions for the prediction and update of potential probability density, which is not the first-order approximate assumption of the number of targets and has higher accuracy in estimating them. However, as shown in the formula, the Gaussian mixture CPHD filter also has calculation problems related to the increase in the number of Gaussian components over time:

(19) $$\left(J_{k-1}(1+J_{\beta ,k})+J_{\gamma ,k})(1+\left|Z_{k}\right|)=O(J_{k-1}\left|Z_{k}\right|\right)$$

Ba-Ngu and Vo [36] processed the predicted Gaussian component of the next step in their study; gave the closed recursion of the mean, covariance, and weight of the Gaussian component of the posterior strength after the GM-CPHD processing; and extended the GM-CPHD filter to apply it to the dynamics of mild nonlinear targets. The main reason for this is that the GM-CPHD filter still needed to undergo “Pruning” and “merging” in this study. “Pruning” refers to the prediction with a high preset threshold or the prediction with a certain number of high weights. “Merging” means that closely spaced Gaussian components are considered to be the same components, so as to be merged together.

Let us set the maximum allowable number of truncation threshold T, merge threshold U and Gaussian terms $J_{\max}$, repeat the following steps until I=ϕ, at which time $l>J_{\max}$, replace $J_{\max}$ with the Gaussian distribution result with the maximum weight ${\left\{{\tilde{\omega }}_{k}^{\left(i\right)},{\tilde{m}}_{k}^{\left(i\right)},{\tilde{P}}_{k}^{\left(i\right)}\right\}}_{i=1}^{l}$, and take ${\left\{{\tilde{\omega }}_{k}^{\left(i\right)},{\tilde{m}}_{k}^{\left(i\right)},{\tilde{P}}_{k}^{\left(i\right)}\right\}}_{i=1}^{l}$ as the output of the trimmed Gaussian component.

(20) l=l+1

(21) $$j=\arg\underset{i\in I}{\max}\omega_{k}^{\left(i\right)}$$

(22) $$L:=\{i\in I|{\left(m_{k}^{\left(i\right)}-m_{k}^{\left(j\right)}\right)}^{Τ}{\left(P_{k}^{\left(i\right)}\right)}^{-1}(m_{k}^{\left(i\right)}-m_{k}^{\left(j\right)})\leq U\}$$

(23) $${\tilde{\omega }}_{k}^{\left(l\right)}=\sum_{i\in L}\omega_{k}^{\left(i\right)}$$

(24) $${\tilde{m}}_{k}^{\left(l\right)}=\frac{1}{{\tilde{\omega }}_{k}^{\left(l\right)}}\sum_{i\in L}\omega_{k}^{\left(i\right)}x_{k}^{\left(i\right)}$$

(25) $${\tilde{P}}_{k}^{\left(l\right)}=\frac{1}{{\tilde{\omega }}_{k}^{\left(l\right)}}\sum_{i\in L}\omega_{k}^{\left(i\right)}(P_{k}^{\left(i\right)}+({\tilde{m}}_{k}^{\left(l\right)}-m_{k}^{i}){\left({\tilde{m}}_{k}^{\left(l\right)}-m_{k}^{i}\right)}^{Τ})$$

(26) $$I:=\frac{I}{L}$$

**Figure 1:**
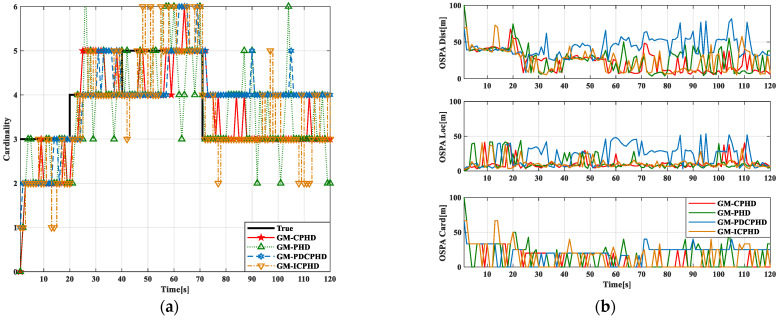
Comparison of filtering effects of multiple filters. (**a**) Comparison of real value detection of multiple filters; (**b**) OSPA error comparison of multiple filters.

In order to detect the filtering effect of the GM-CPHD filter, this study presents different effects on the GM-CPHD, GM-PHD, GMPDCPHD, and GM-ICPHD of CPHD, PHD, PDCPHD, and ICPHD filters under the Gaussian distribution. The filtering effects of various filters are shown in Figure 1. This shows that the effect is relatively stable compared with the CPHD filter, so this study selects the GM-CPHD filter for research and analysis.

## 3. Inverse Covariance Intersection

### 3.1. CI Integration and ICI Integration

Covariance cross-fusion: In two-sensor systems, if the estimation error variance matrix $P_{A},P_{B}$ of the subsystem is known and the cross-covariance $P_{A,CI},P_{B,CI}$ is unknown, the covariance cross-fusion algorithm is as follows:

(27) $$x_{CI}=P_{CΙ}(\omega_{CI}P_{A}^{-1}x_{A}+(1-\omega_{CI})P_{B}^{-1}x_{B})=P_{CΙ}(P_{A,CI}^{-1}x_{A}+P_{B,CI}^{-1}x_{B})$$

(28) $$P_{CI}=(\omega_{CI}P_{A}^{-1}+(1-\omega_{CI})P_{B}^{-1}{)}^{-1}=(P_{A,CI}^{-1}+P_{B,CI}^{-1}{)}^{-1}$$

(29) $$P_{A,CI}^{-1}≜\omega_{CI}P_{A}^{-1}$$

(30) $$P_{B,CI}^{-1}≜(1-\omega_{CI})P_{B}^{-1}$$

The ω∈[0,1] minimization performance index is as follows:

(31) $$\min J=\underset{\omega \in [0,1]}{\min}trP_{CI}=\underset{\omega \in [0,1]}{\min}tr\left\{{\left[\omega {\left(P_{A}\right)}^{-1}+(1-\omega ){(P_{B})}^{-1}\right]}^{-1}\right\}$$

Inverse covariance intersection fusion: In the two-sensor systems, if the estimation error variance matrix $P_{A},P_{B}$ of the subsystem is known and the cross-covariance $P_{A,CI},P_{B,CI}$ is unknown, the inverse covariance cross-fusion algorithm is as follows:

(32) $$x_{ICI}=K_{ICΙ}x_{A}+L_{ICΙ}x_{B}$$

(33) $$P_{ICI}=P_{A,ICI}^{-1}+P_{B,ICI}^{-1}-{(\omega_{ICI}P_{A}+(1-\omega_{ICI})P_{B})}^{-1}$$

(34) $$K_{ICI}=P_{ICI(}P_{A}^{-1}-\omega_{ICI}{(\omega_{ICI}P_{A}+(1-\omega_{ICI})P_{B})}^{-1}$$

(35) $$L_{ICI}=P_{ICI(}P_{B}^{-1}-(1-\omega_{ICI}){(\omega_{ICI}P_{A}+(1-\omega_{ICI})P_{B})}^{-1}$$

The ω∈[0,1] minimization performance index is as follows:

(36) $$\min J=\underset{\omega \in [0,1]}{\min}trP_{ICI}=\underset{\omega \in [0,1]}{\min}tr\left\{{\left[{\left(P_{A}\right)}^{-1}+{\left(P_{B}\right)}^{-1}-(\omega P_{A}+(1-\omega ){(P_{B})}^{-1}\right]}^{-1}\right\}$$

Moreover, for any $\omega_{CI}\in [0,1]$, there is always a $\omega_{ICI}=1-\omega_{CI}$, so that $P_{ICI}[\omega_{ICI}]\leq P_{CI}[\omega_{CI}]$.

### 3.2. PICI Fusion Algorithm

**Figure 2:**
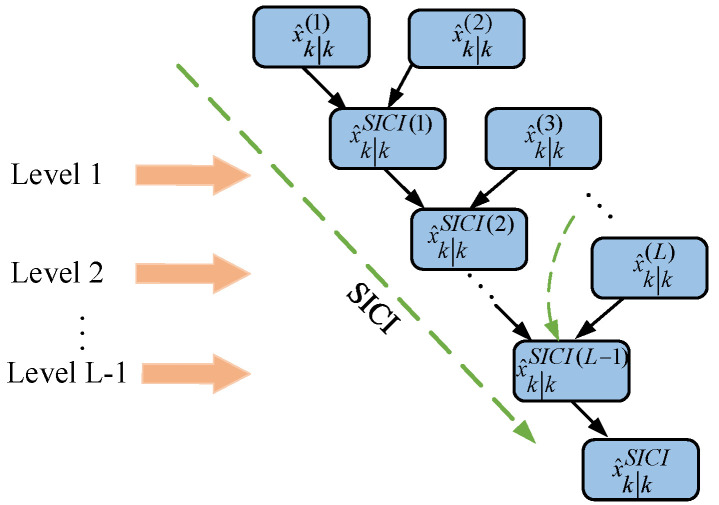
SICI fusion algorithm.

When there are many local estimators, the calculation of convex combination coefficients is complex, which limits the real-time performance of the system’s level calculation. The sequential inverse covariance intersection (SICI) multisensor fusion algorithm consists of a (L − 1) dual-sensor system based on the ICI method. The SICI fusion algorithm is shown in Figure 2 [17].

**Figure 3:**
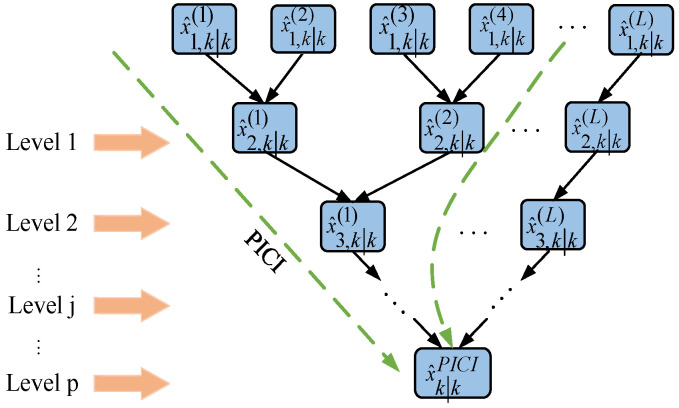
PICI fusion algorithm.

When there are multiple sensors in the system, the SICI fusion algorithm needs to perform L − 1 double fusion, which results in a significant amount of running time. If the number of sensors L remains unchanged, the number of two-sensor ICI fusion devices in layer j will decrease exponentially with the increase in j. Therefore, when the number of sensors L is particularly high, the parallel inverse covariance intersection (PICI) algorithm can save a significant amount of computing time. In order to save system operation time, the parallel covariance intersection fusion algorithm is used. The PICI fusion algorithm is shown in Figure 3.

In the PICI multisensor fusion algorithm, the fusion estimation of ${\hat{x}}_{k|k}^{\left(i\right)}(i,i+1,\cdots ,L)$ at different levels is determined by the subsystem estimation ${\hat{x}}_{k|k,2i-1}^{\left(ICI,j-1\right)},{\hat{x}}_{k|k,2i}^{\left(ICI,j-1\right)}$ and the subsystem variance $P_{ICI,2i-1}^{\left(j-1\right)},P_{ICI,2i}^{\left(j-1\right)}$ of the two-sensors ICI fusion at the upper level, so the PICI fusion algorithm is as follows:

(37) $${\hat{x}}_{k|k,i}^{\left(ICI,j\right)}=K_{k,i}^{\left(j\right)}{\hat{x}}_{k|k,2i-1}^{\left(ICI,j-1\right)}+L_{k}^{\left(j\right)}{\hat{x}}_{k|k,2i}^{\left(ICI,j-1\right)}$$

(38) $$P_{ICI}^{\left(j\right)}={\left[{\left(P_{ICI,2i-1}^{\left(j-1\right)}\right)}^{-1}+{\left(P_{ICI,2i}^{\left(j-1\right)}\right)}^{-1}-{\left(\omega_{i}^{\left(j\right)}P_{ICI,2i-1}^{\left(j-1\right)}+(1-\omega_{i}^{\left(j\right)})P_{ICI,2i}^{\left(j-1\right)}\right)}^{-1}\right]}^{-1}$$

(39) $$K_{K}^{\left(j\right)}=P_{ICI,i}^{\left(j\right)}{\left[{\left(P_{ICI,2i-1}^{\left(j-1\right)}\right)}^{-1}-\omega_{i}^{\left(j\right)}(\omega_{i}^{\left(j\right)}P_{ICI,2i-1}^{\left(j-1\right)})+(1-\omega_{i}^{\left(j\right)})P_{ICI,2i}^{\left(j-1\right)}\right)}^{-1}]$$

(40) Lk,i(j)=PICI,i(j)[(PICI,2i(i−1))−1−(1−ω)i(j)(ωPi(j)ICI,2i−1(i−1)+(1−ω)i(j)PICI,2i(i−1))−1]

where $i=1,2,\cdots ,L_{j},j=1,2,\cdots L_{p}$, $L_{j},L_{p}$ are the number of filters in layer *j* and the number of final layers, respectively.

For this reason, the initial value can be considered as follows:

(41) $${\hat{x}}_{k|k,i}^{\left(ICI,1\right)}={\hat{x}}_{k|k}^{\left(i\right)},P_{ICI,i}^{\left(1\right)}=P_{k|k}^{\left(i\right)},L_{1}=L$$

The ω∈[0,1] minimization performance index is as follows:

(42) $$\min J=\underset{\omega_{1}\in [0,1]}{\min}trP_{ICI}=\underset{\omega_{1}\in [0,1]}{\min}tr\left\{{\left[{\left(P_{k|k}^{\left(1\right)}\right)}^{-1}+{\left(P_{k|k}^{\left(2\right)}\right)}^{-1}-(\omega_{1}P_{k|k}^{\left(1\right)}+(1-\omega_{1}){(P_{k|k}^{\left(2\right)})}^{-1}\right]}^{-1}\right\}$$

(43) $$\begin{array}{l} \min J=\underset{\omega_{i}\in [0,1]}{\min}trP_{ICI,i}^{\left(j\right)}\\ =\underset{\omega_{{}_{i}}^{j}\in [0,1]}{\min}tr\left\{{\left[{\left(P_{ICI,2i-1}^{\left(j-1\right)}\right)}^{-1}+{\left(P_{ICI,2i}^{\left(j-1\right)}\right)}^{-1}-(\omega_{i}^{\left(j\right)}P_{ICI,2i+1}^{\left(j-1\right)}+(1-\omega_{i}^{\left(j\right)}){(P_{ICI,2i}^{\left(j-1\right)})}^{-1}\right]}^{-1}\right\} \end{array}$$

**Figure 4:**
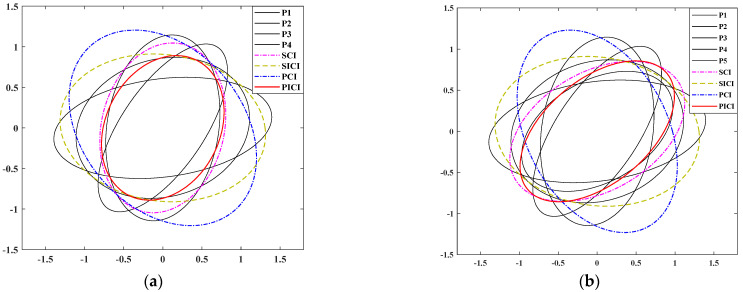
Comparison results of different covariance intersection fusion algorithms. (**a**) L = 4; (**b**) L = 5.

The effects of the SCI, PCI, SICI, and PICI algorithms are compared by covariance ellipse verification with L = 4 and L = 5 to verify the effectiveness of the PICI fusion algorithm. The covariance ellipse values under L = 4 and L = 5 are shown in Table 1, and the comparison results of different covariance intersection fusion algorithms are shown in Figure 4. This shows that the covariance ellipse of the ICI algorithm is visibly higher than the fusion precision of the CI algorithm regardless of whether L = 4 or L = 5, and the fusion effect of the PICI fusion algorithm is stronger than other algorithms.

## 4. PICI-GM-CPHD

Since ICI has not yet been extended to a multisensor multitarget system, WOO JUNG PARK [26] proposed covariance inflation naïve fusion in order to apply ICI to multisensor multitarget systems in the relevant research, and changed the original structure of ICI to the following:

(44) $$x_{ICI}=K_{ICΙ}x_{A}+L_{ICΙ}x_{B}$$

(45) $$P_{ICI}={\left(P_{A}^{-1}+P_{B}^{-1}-{(\omega_{ICI}P_{A}+(1-\omega_{ICI})P_{B})}^{-1}\right)}^{-1}={\left(P_{A,ICI}^{-1}+P_{B,ICI}^{-1}\right)}^{-1}$$

(46) $$P_{A,ICI}^{-1}≜P_{A}^{-1}-\omega_{ICI}{(\omega_{ICI}P_{A}+(1-\omega_{ICI})P_{B})}^{-1}$$

(47) $$P_{B,ICI}^{-1}≜P_{B}^{-1}-(1-\omega_{ICI}){(\omega_{ICI}P_{A}+(1-\omega_{ICI})P_{B})}^{-1}$$

It can also be verified that $x^{Τ}P_{A,CI}x>0,x^{Τ}P_{B,CI}x>0$, and $P_{A,CI},P_{B,CI}$ are positive definite, namely

(48) $$x^{Τ}P_{A,ICI}x=x^{Τ}P_{A}x+\frac{\omega }{1-\omega }x^{Τ}P_{B}^{-1}P_{A}x=x^{Τ}P_{A}x+\frac{\omega }{1-\omega }y^{Τ}P_{B}^{-1}y>0$$

(49) $$x^{Τ}P_{B,ICI}x=x^{Τ}P_{B}x+\frac{\omega }{1-\omega }x^{Τ}P_{A}^{-1}P_{B}x=x^{Τ}P_{B}x+\frac{\omega }{1-\omega }y^{Τ}P_{A}^{-1}y>0$$

Similarly, by combining the above methods, this study describes the parallel inverse covariance intersection based on the covariance inflation naïve fusion and rewrites PICI as follows:

(50) $$x_{PICI}=K_{PICΙ}x_{A}^{j-1}+L_{PICΙ}x_{B}^{j-1}$$

(51) $$\begin{array}{ll} P_{PICI} & ={\left(P_{A}^{j-1}\right)}^{-1}+{\left(P_{Β}^{j-1}\right)}^{-1}-(\omega_{PICI}P_{A}^{j-1}+(1-\omega_{PICI})P_{Β}^{j-1})-1{)}^{-1}\\ & ={\left({\left(P_{A,PICI}^{j-1}\right)}^{-1}+{\left(P_{Β,PICI}^{j-1}\right)}^{-1}\right)}^{-1} \end{array}$$

The above (Formulas (50) and (51)) shows that the fusion result of the lower level of the PICI algorithm is determined by the fusion result of the upper level. For this reason, the PICI and ICI algorithms can be different in the determination of $P_{A}^{-1},P_{B}^{-1}$. The PICI algorithm needs to be divided into $P_{L_{P},k|k}^{A(m)},P_{L_{P},k|k}^{B(m)}$ types at different levels. When the number of sensors *L* is even, then $m=\frac{L}{2}$; when the number of sensors *L* is odd, then $m=\frac{L-1}{2}$.

(52) $${\left(P_{A,ICI}^{\left(y\right)}\right)}^{-1}≜{\left(P_{A}^{\left(y\right)}\right)}^{-1}-\omega_{ICI}{(\omega_{ICI}P_{A}^{\left(y\right)}+(1-\omega_{ICI})P_{B}^{\left(y\right)})}^{-1}$$

(53) $${\left(P_{B,ICI}^{\left(y\right)}\right)}^{-1}≜{\left(P_{B}^{\left(y\right)}\right)}^{-1}-\omega_{ICI}{(\omega_{ICI}P_{B}^{\left(y\right)}+(1-\omega_{ICI})P_{A}^{\left(y\right)})}^{-1}$$

where y=1,2,⋯,m.

**Figure 5:**
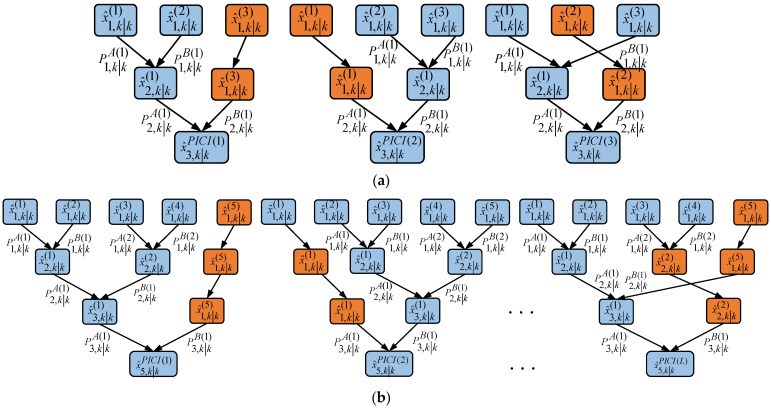
Comparison results of different covariance intersection fusion algorithms; (**a**) 3-sensor PICI fusion, (**b**) 5-sensor PICI fusion.

It is also considered that the PICI algorithm has multiple integration situations, as shown in Figure 5. Therefore, the results of different sensor combination modes can be set as follows.

The weighting matrix is represented by

(54) $$M_{i}^{PICI}=M_{i(L)}^{PICI}\cdots M_{i(2)}^{PICI}M_{i(1)}^{PICI}$$

(55) $$N_{i}^{PICI}=N_{i(L)}^{PICI}\cdots N_{i(2)}^{PICI}N_{i(1)}^{PICI}$$

Therefore, PICI-GM-CPHD algorithm is shown in Algorithm 1:
**Algorithm 1:** PICI-GM-CPHD filtering algorithm flow1. Calculate GM-CPHD filter, calculate GM-GPHD prediction, update, “pruning”, and “merging”;2. For l=1:L (*L* is number of sensors);3. Calculate PICI fusion weight using covariance intersection;4. Replace the covariance of the probability density a of $P_{A}^{\left(y\right)},P_{B}^{\left(y\right)}$ single sensor with $\left\{ \begin{array}{l} P_{A,ICI}^{\left(y\right)}=P_{A}^{\left(y\right)}+\frac{\omega_{ICI}}{1-\omega_{ICI}}P_{A}^{\left(y\right)}{\left(P_{B}^{\left(y\right)}\right)}^{-1}P_{A}^{\left(y\right)}\\ P_{B,ICI}^{\left(y\right)}=P_{B}^{\left(y\right)}+\frac{\omega_{ICI}}{1-\omega_{ICI}}P_{B}^{\left(y\right)}{\left(P_{A}^{\left(y\right)}\right)}^{-1}P_{B}^{\left(y\right)} \end{array}\right.$;5. Different calculations $P_{L_{P},k|k}^{A(m)},P_{L_{P},k|k}^{B(m)}$;6. Calculate the fusion result of the next level according to the fusion result of the previous level;7. Determine the PICI fusion results of multiple sensors according to Formulas (50) and (51);8. Calculate GM covariance by naïve fusion of covariance expansion (52)–(54);9. Modify and improve PICI-GM-CPHD through “pruning” and “merging”;10. Calculate different PICI-GM-CPHD weighting matrices;11. End of algorithm;12. Estimate extraction.

## 5. Modeling and Simulation

Since both linear and nonlinear models are involved in this study, a constant velocity model (CV) is used for all linear Gaussian models. The constant turn rate and velocity (CTRV) model is used for all nonlinear models.

The linear model CV model is $CV(x)={\left(x\;\vartheta \;y\;v\right)}^{⊤}$, wherein the four state variables are x as the abscissa, ϑ as the angle with the x-axis (counterclockwise is positive), y as the ordinate, and v as the linear velocity.

(56) $$F_{k}=\left[\begin{array}{cc} I_{2} & \Delta I_{2}\\ O_{2} & I_{2} \end{array}\right]$$

(57) $$Q_{K}=\sigma_{v}^{2}\left[\begin{array}{cc} \frac{\Delta^{4}}{4}I_{2} & \frac{\Delta^{3}}{2}I_{2}\\ \frac{\Delta^{3}}{2}I_{2} & \Delta^{2}I_{2} \end{array}\right]$$

(58) $$H_{k}=[\begin{array}{cc} I_{2} & O_{2} \end{array}],\;R_{k}=\sigma_{\varepsilon }^{2}I_{2}$$

The nonlinear *CTRV* model is $CTRV(x)={\left(x\;\vartheta \;y\;\psi \;\dot{\psi }\right)}^{⊤}$, wherein the five variables are x as abscissa, y as ordinate, v as linear velocity, ψ as yaw angle (counterclockwise is positive with the included angle), and $\dot{\psi }$ as angular velocity.

(59) $$x_{k}=\left[\begin{array}{ccccc} 1 & \frac{\sin\Omega T}{\Omega } & 0 & -\frac{1-\cos\Omega T}{\Omega } & 0\\ 0 & \cos\Omega T & 0 & -\sin\Omega T & 0\\ 0 & \frac{1-\cos\Omega T}{\Omega } & 1 & \frac{\sin\Omega T}{\Omega } & 0\\ 0 & \sin\Omega T & 0 & \cos\Omega T & 0\\ 0 & 0 & 0 & 0 & 1 \end{array}\right]x_{k-1}+v_{k}$$

The measurement noise is

(60) $$wk∼N(0,R),R=diag[\sigma_{r}^{2}\sigma_{\theta }^{2}]$$

(61) $$Q=diag[q_{1}M\;q_{1}M\;q_{1}T]$$

The GM-CPHD filter is set to have a detection probability of 0.9 and a survival probability of 0.99. The average Poisson rate of the moving target’s uniform clutter is 5, and the birth density is (±800 m, ±800 m). All simulations are completed by 120 Monte Carlo experiments. The truncation threshold, combined threshold, and the maximum allowable number of Gaussian terms are set in the GM-CPHD filter. The tracking performance of the moving targets is measured by the OSPA distance. We set the cut-off parameter and order parameter as c = 100 and p = 1, respectively.

### 5.1. Linear Gaussian Measurement Model

**Figure 6:**
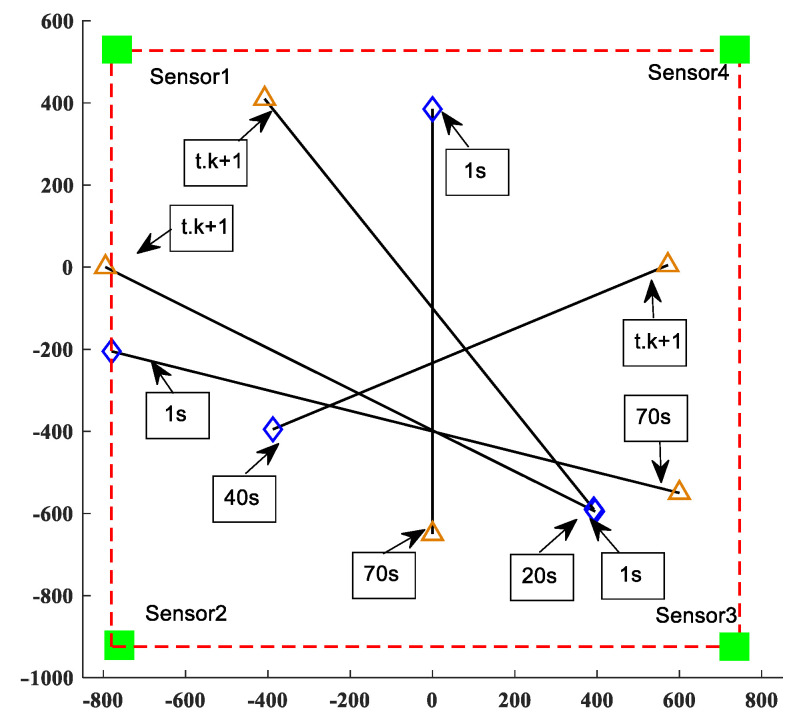
Linear CV model’s moving target trajectory.

**Figure 7:**
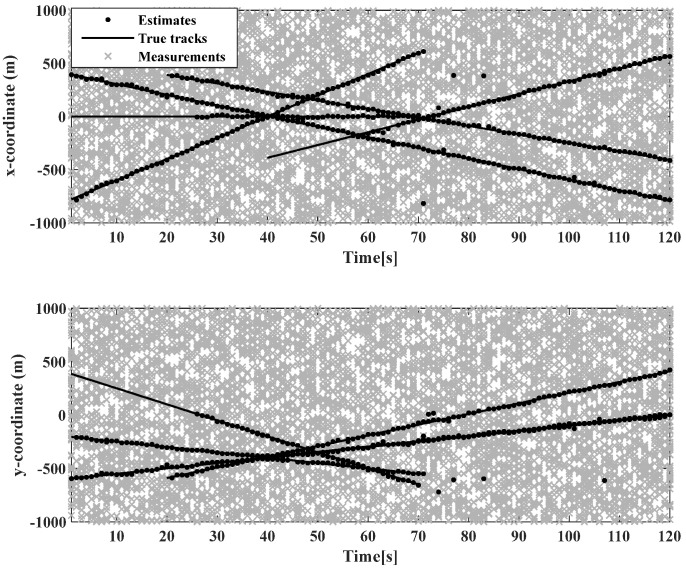
Real trajectories, measurements, and estimates in Cartesian coordinates in linear models.

The initial state of the linear Gaussian measurement model target is shown in Table 2. As shown in Figure 6, the moving target trajectory of the linear CV model is shown, while Figure 7 shows the real trajectory, measured values, and estimated values in the Cartesian coordinate system of the linear model. This shows that the birth time of different targets, as well as the state of moving targets detected by the sensors, is different.

**Figure 8:**
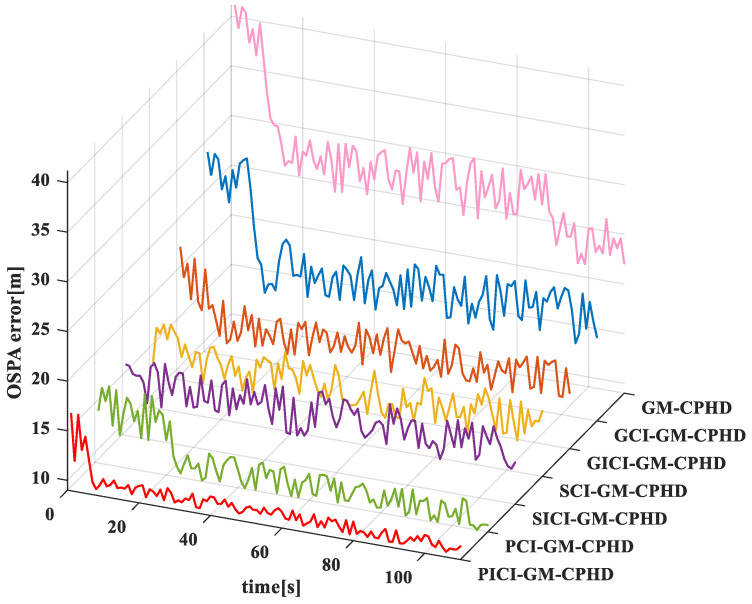
Comparison of OSPA distance errors of several algorithms in linear models.

In order to compare the effects of various algorithms, the comparison results of the GM-CPHD (single sensor 1), GCI-GM-CPHD, GICI-GM-CPHD, SCI-GM-CPHD, SICI-GM-CPHD, PCI-GM-CPHD, and PICI-GM-CPHD algorithms are evaluated, as shown in Figure 8. From the comparison and results of the OSPA distance error of several algorithms in the linear model, it can be seen that the distance error of the GM-CPHD and GCI-GM-CPHD algorithms in the first 20 s is large and then tends to be stable. The OSPA distance error of the SCI-GM-CPHD, SICI-GM-CPHD, SICI-GM-CPHD, PICI-GM-CPHD, and PICI-GM-CPHD centralized algorithms is relatively stable in operation, and the main reason for this is related to the appearing and disappearing frame of the moving objects. Compared with the PICI-GM-CPHD algorithm, the OSPA distance comprises fewer errors in the linear model.

### 5.2. Nonlinear Gaussian Measurement Model

**Figure 9:**
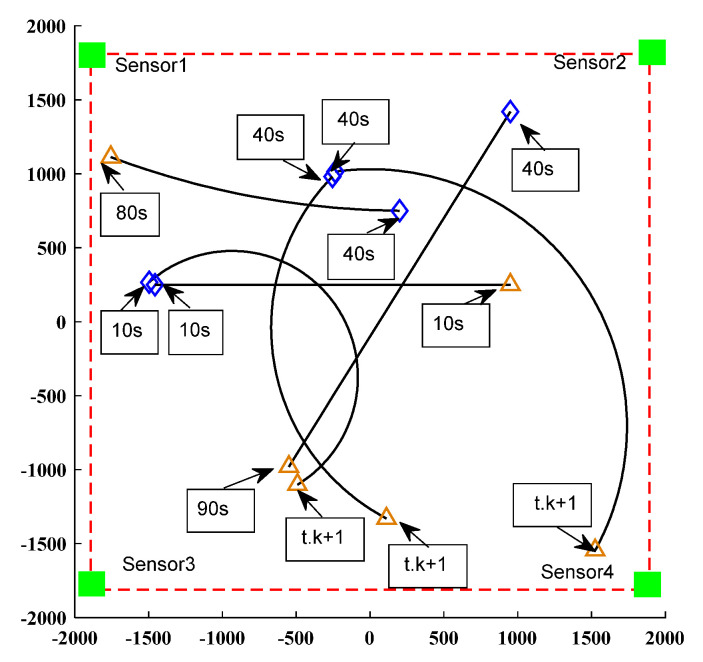
Nonlinear CV model’s moving target trajectory.

**Figure 10:**
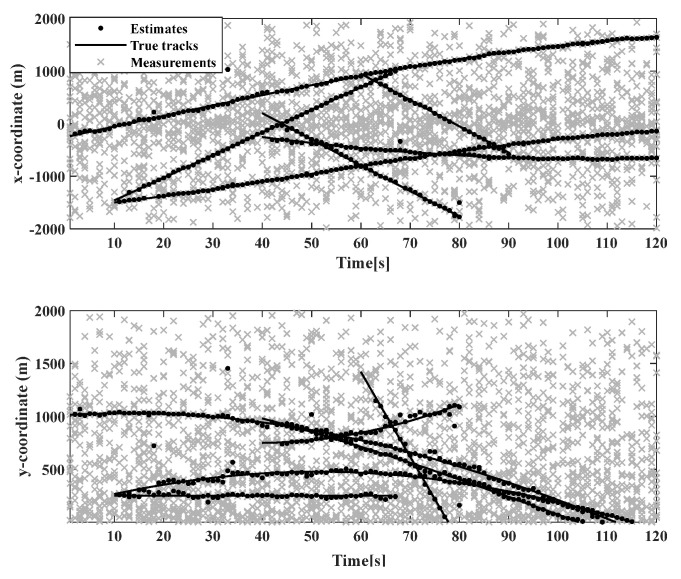
Real trajectories, measurements, and estimates in Cartesian coordinates in nonlinear models.

The initial state of the target of the nonlinear linear Gaussian measurement model is shown in Table 3. In Figure 9, the trajectory of the moving target in the nonlinear model CV model is shown, while Figure 10 shows the real trajectory, measured values, and estimated values in the Cartesian coordinate system in the nonlinear model. This shows that the birth time of different targets, as well as the state of moving targets detected by the sensors, is different.

**Figure 11:**
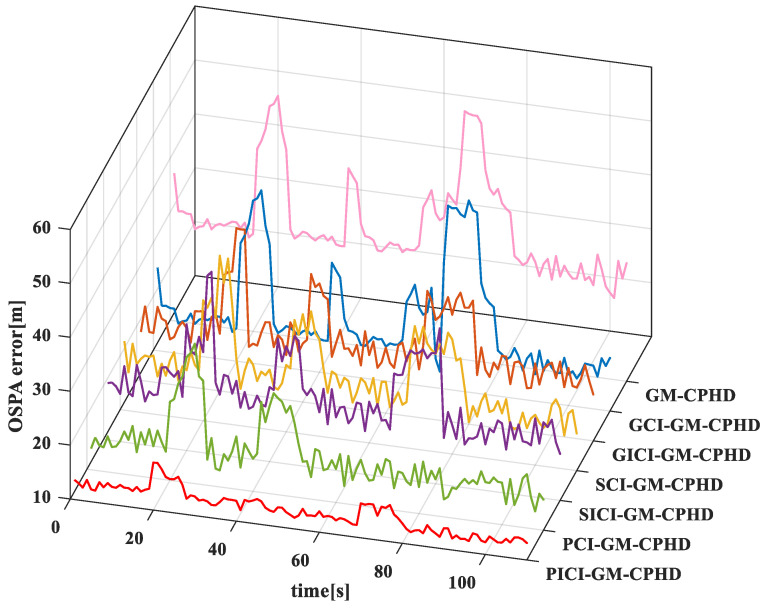
Comparison of OSPA distance errors of several algorithms in nonlinear models.

The OSPA distance error of the GM-CPHD (single sensor 1), GCI-GM-CPHD, GICI-GM-CPHD, SCI-GM-CPHD, SICI-GM-CPHD, PCI-GM-CPHD, and PICI-GM-CPHD algorithms in the nonlinear model are compared, as shown in Figure 11. From the OSPA distance error comparison and results of several algorithms in the nonlinear model, it can be seen that the occurrence of OSPA distance errors in the nonlinear model is lesser than that in the PICI-GM-CPHD algorithm. However, in the nonlinear model, the OSPA distance errors of the GICI-GM-CPHD, SCI-GM-CPHD, SICI-GM-CPHD, PCI-GM-CPHD, and PICI-GM-CPHD algorithms are relatively concentrated, and the differences between the OSPA distance errors are not significant. This shows that the effect of the above centralized algorithms in the nonlinear model is not as satisfactory as that in the linear model.

**Figure 12:**
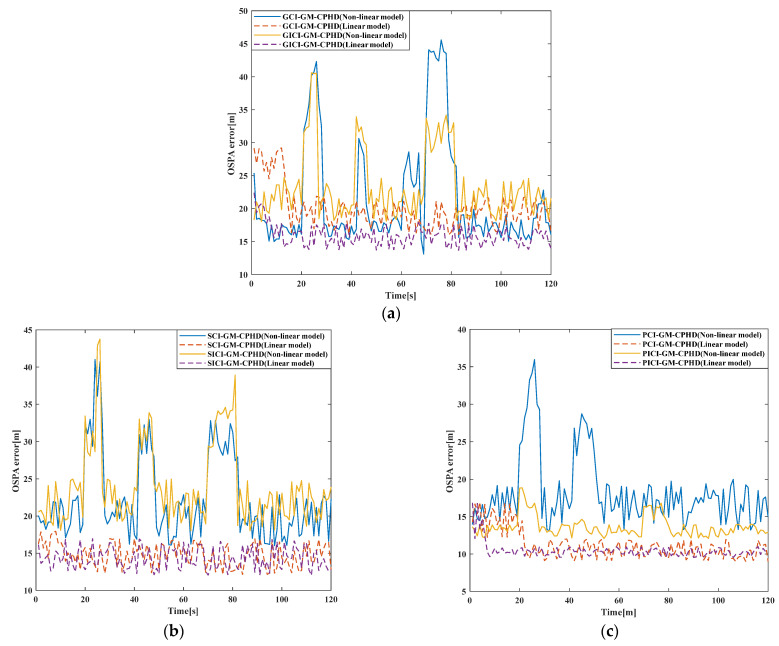
Comparison of OSPA distance errors of several algorithms in nonlinear models. (**a**) GCI, GICI; (**b**) SCI, SICI; (**c**) PCI, PICI.

In order to compare the effects of different fusion algorithms in different models, the OSPA distance errors of different fusion algorithms in different models are compared. Figure 12 shows the comparison of OSPA distance errors of several algorithms in nonlinear models. It can be seen from the results that the occurrence of OSPA distance errors of the above models, in the case of the linear model, is significantly lesser than that in the case of the nonlinear model, which shows that the above algorithms are more suitable for a linear model. In the comparison between the linear model and the nonlinear model, the difference between the GCI-GM-CPHD and GICI-GM-CPHD algorithms is not significant, and the difference between the SCI-GM-CPHD and SICI-GM-CPHD algorithms is not significant, while the difference between the PCI-GM-CPHD and PICI-GM-CPHD algorithms is small. Therefore, the fusion method of the PCI-GM-CPHD and PICI-GM-CPHD algorithms can be more applicable to different models. In addition, the PCI-GM-CPHD and PICI-GM-CPHD algorithms have the minimum OSPA distance errors in the linear model, indicating that these two algorithms have the best effect in the linear model.

Table 4 shows the comparison results of the algorithm’s running time. This shows that the resulting Monte Carlo calculation time used by the GM-CPHD algorithm is relatively short among the above algorithms. The main reason for this is that the GM-CPHD algorithm mainly aims at the filtering effect of a single sensor. The running time of the ICI filtering algorithm is higher than that of the CI filtering algorithm; compared with the GICI-GM-CPHD, SICI-GM-CPHD, PICI-GM-CPHD, and PICI-GM-CPHD algorithms, the PICI-GM-CPHD algorithm has a lower computing time, which is similar to that between the GCI-GM-CPHD and GICI-GM-CPHD algorithms. Compared with the SICI-GM-CPHD algorithm, the PICI-GM-CPHD algorithm has a longer computing time. The PICI-GM-CPHD algorithm can save a significant amount of computing time and is more suitable for distributed sensor fusion systems with more sensors.

## 6. Conclusions

In this study, a GM-CPHD filter based on PICI is proposed, and the effectiveness of the algorithm is verified by simulation with linear and nonlinear Gaussian measurement models. In order to apply PICI to the GM-CPHD filter in this research, the original structure of the ICI was changed, the PICI fusion mode was reconstructed, and this was applied to the nonlinear motion model. The simulation experiment compares the GM-CPHD (single sensor 1), GCI-GM-CPHD, GICI-GM-CPHD, SCI-GM-CPHD, SICI-GM-CPHD, SICI-GM-CPHD, PICI-GM-CPHD, PICI-GM-CPHD, and PICI-GM-CPHD algorithms. The simulation results effectively prove that the PICI-GM-CPHD fusion tracking algorithm has a high estimation accuracy. From the comparison results of the Monte Carlo calculation time of various algorithms in different models, it was shown that the PICI-GM-CPHD algorithm can save a significant amount of calculation time and is more suitable for distributed fusion with a large number of sensors.

## Figures and Tables

**Table 1:** Value of covariance ellipse under L = 4 and L = 5.

	L = 4	L = 5
P1	[0.6855, 0.5733; 0.5733, 1.0707]	[0.6855, 0.5733; 0.5733, 1.0707]
P2	[1.9433, 0.1872; 0.1872, 0.3895]	[1.9433, 0.1872; 0.1872, 0.3895]
P3	[1.2310, 0.1352; 0.1352, 0.7560]	[1.2310, 0.1352; 0.1352, 0.7560]
P4	[0.5421, 0.1753; 0.1753, 1.3345]	[0.5421, 0.1753; 0.1753, 1.3345]
P5		[1.5642, 0.8236; 0.8236, 0.8837]

**Table 2:** Initial state of linear Gaussian measurement model target.

Target	Initial State	Appearing Frame	Disappearing Frame
1	[0; 0; 400; −15; 0]	1	70
2	[400; −10; −600; 5; 0]	1	truth.K + 1
3	[−800; 20; −200; −5; 0]	1	70
4	[400; −8; −600; 10; 0]	20	truth.K + 1
5	[−400; 12; −400; 5; 0]	40	truth.K + 1

**Table 3:** Initial state of nonlinear Gaussian measurement model target.

Target	Initial State	Appearing Frame	Disappearing Frame
1	[−250 − 5.8857; 20; 1000 + 11.4102; 3; −wturn/3]	1	truth.K + 1
2	[−1500 − 7.3806; 11; 250 + 6.7993; 10; −wturn/2]	10	truth.K + 1
3	[−1500; 43; 250; 0; 0]	10	66
4	[−250 + 7.3806; v12; 1000 − 6.7993; −12; wturn/3]	40	truth.K + 1
5	[250; −50; 750; 0; −wturn/4]	40	80
6	[1000; −50; 1500; −80; 0]	60	90

**Table 4:** Comparison results of algorithm running time.

Target	GM- CPHD	GCI- GM-CPHD	GICI- GM-CPHD	SCI- GM-CPHD	SICI- GM-CPHD	PCI- GM-CPHD	PICI- GM-CPHD
Linear model	42.54	93.42	115.37	165.32	190.36	129.53	112.58
Nonlinear model	42.52	95.15	115.34	172.34	195.48	132.56	114.12
Average	42.53	94.285	115.355	168.83	192.92	131.045	113.35

## Data Availability

Not applicable.
